# Toward Standardized Protocols: Determining Optimal Stimulation Volumes for 5 Hz Repetitive Peripheral Magnetic Stimulation (rPMS) of the Tibial Nerve—A Controlled Exploratory Study

**DOI:** 10.3390/brainsci16010100

**Published:** 2026-01-17

**Authors:** Volker R. Zschorlich, Dirk Büsch, Sarah Schulte, Fengxue Qi, Jörg Schorer

**Affiliations:** 1 Institute of Sports Science, Faculty of Philosophy, University of Rostock, Am Waldessaum, 18057 Rostock, Germany; 2 Institute of Sport Science, Faculty IV, Carl von Ossietzky University Oldenburg, Ammerländer Heerstraße 114-118, 26129 Oldenburg, Germany; dirk.buesch@uni-oldenburg.de (D.B.); sarah.schulte1@uni-oldenburg.de (S.S.); joerg.schorer@uni-oldenburg.de (J.S.); 3 Department Aging of Individuals and Society, Faculty of Interdisciplinary Research, University of Rostock, Gehlsheimer Str. 20, 18051 Rostock, Germany; 4 Sports, Exercise and Brain Sciences Laboratory, Beijing Sport University, Beijing 100084, China; fengxue.qi@hotmail.com

**Keywords:** EMG, spasticity, muscle tone, compound muscle action potential, Achilles tendon reflex, neuroplasticity, LTD, dose-response, physical therapy, rehabilitation

## Abstract

**Background**: Repetitive peripheral magnetic stimulation (rPMS) has emerged as a promising non-invasive treatment modality for reducing muscle hypertonus and spasticity. However, standardized protocols regarding stimulation parameters, particularly the number of stimuli required to achieve therapeutic effects, remain largely undefined. **Methods**: In an exploratory study, seventeen healthy participants (15 male, 2 female) underwent progressive rPMS treatments at 5 Hz frequency with incrementally increasing stimulus counts (105, 210, 315, 420, and 840 stimuli). Seventeen participants served as controls (11 male, 6 female) receiving sham stimulation. Achilles tendon reflexes were elicited using a computer-controlled reflex hammer, and compound muscle action potential (CMAP) peak-to-peak amplitudes were recorded via surface electromyography before and immediately after each stimulation session. **Results**: The overall repeated-measures ANOVA indicated a significant main effect (*F*(5, 80) = 4.98, *p* = 0.001, η^2^_p_ = 0.237). All rPMS treatments produced significant reductions in CMAP amplitudes compared to baseline (*p* < 0.05). No progressive dose-dependent relationship was observed between stimulus count and response magnitude, suggesting a threshold effect rather than progressive inhibition. Control group showed no significant changes (*p* ≤ 0.56). **Conclusions**: Low-frequency (5 Hz) rPMS produces rapid inhibitory effects on spinal reflex circuits with onset after as few as 105 stimuli. These findings indicate that treatment effects can be achieved with substantially fewer stimuli than previously assumed. Further research is needed to identify parameters capable of achieving greater reflex suppression.

## 1. Introduction

Skeletal muscle hypertonus represents a prevalent clinical condition associated with considerable discomfort and functional impairment. While pathological hypertonicity is characteristic of neurological conditions such as spasticity and rigidity, elevated muscle tone also occurs in healthy individuals following intensive resistance training or eccentric exercise [1].

Several studies have documented successful reduction in spasticity and muscle tone or muscle pain [2] through low-frequency repetitive peripheral magnetic stimulation (rPMS) protocols. The efficacy of using 25 Hz stimulation [3] was demonstrated in multiple sclerosis patients, while others [4] employed 20 Hz protocols in healthy subjects. Our own investigations have utilized 15 Hz stimulation [5] and 5 Hz protocols [6]. Furthermore, theta-burst stimulation with 5 Hz repetition frequency [7] was administered. Despite the promising results of rPMS, fundamental questions regarding the mechanisms of muscle tone modulation, the measurement of spasticity and the optimal stimulation parameters remain largely unresolved [8,9]. A recent systematic review evaluated the effects of rPMS (alone or combined with rTMS) on upper limb motor function and activities of daily living in post-stroke patients [10]. A further review paper evaluated the effects of repetitive peripheral magnetic stimulation (rPMS) on upper limb motor recovery in post-stroke patients [11]. A critical gap in the literature concerns the minimal effective stimulation volume and whether a dose–response relationship exists for rPMS effects. Current rPMS research faces a significant methodological challenge: the absence of standardized protocols regarding key stimulation parameters, including stimulation intensity, often determined by motor threshold or subjective tolerance. Furthermore, the extent of stimulation (number of stimuli per session) is of great importance for the treatment and reported protocols vary from several hundred to several thousand pulses. However, the temporal dynamics and dose–response characteristics of this effect remain unexplored [12]. Specifically, these are unknown: What is the minimal number of stimuli required to produce measurable effects? And is there a progressive relationship between stimulus count and response magnitude? Understanding the different stimulation parameters is essential for developing efficient, evidence-based treatment protocols. The early occurrence of effects in the form of reduced compound muscle action potential (CMAP) amplitude has not been systematically investigated. The primary objective of this study was, therefore, to systematically investigate minimal and optimal stimulation volumes of low-frequency (5 Hz) rPMS applied to the tibial nerve, using CMAP amplitude as a quantitative outcome measure of reflex excitability [13].

## 2. Materials and Methods

### 2.1. Participants

Thirty-four healthy volunteers participated in this study. Participants blinded to hypothesis were randomly assigned to groups (computer-generated sequence) and included both regularly trained athletes and sports students. The experimental group consisted of seventeen participants (mean age 26 ± 3.9 years; height 178.5 ± 7.3 cm; weight 76.7 ± 9.8 kg), while the sham group comprised seventeen participants (28.6 ± 4.9 years; height 178.5 ± 9.8 cm; weight 75.1 ± 11.1 kg). Exclusion criteria included neurological disorders, epilepsy, metallic implants, or any contraindications to magnetic stimulation. All testing was performed on the left leg.

### 2.2. Study Design and Ethical Approval

This study was designed as an exploratory pilot investigation to assess the effects of repetitive peripheral magnetic stimulation according to the CONSORT guidelines. The primary focus was placed on longitudinal changes within the treatment group across multiple measurement time points. A control group was included to provide complementary reference data; however, this sham stimulated group was not assessed at all across the measurement time points. Consequently, comparisons between the treatment and sham group were considered exploratory in nature (no formal sample size calculation), and no fully balanced between-group analyses were intended.

**Figure 1:**
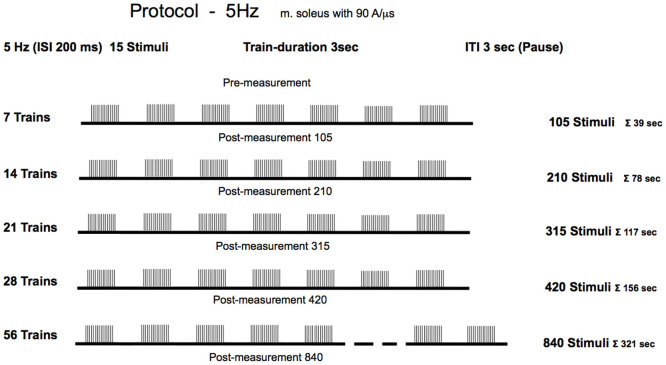
To investigate the dose–response relationship of rPMS, participants underwent a pre-measurement of reflex responses (no stimulation), followed by rPMS. Stimulation was delivered at a frequency of 5 Hz, corresponding to five pulses per second with an inter-stimulus interval of 200 ms. Each train consisted of 15 stimuli, and consecutive trains were separated by 3 s rest intervals. Participants received progressive stimulation sessions with increasing total numbers of stimuli.

The treatment group received active 5 Hz rPMS, while the sham group received sub-threshold stimulation to control for placebo effects, positioning effects, and natural temporal fluctuations in reflex excitability. Reflex measurements were obtained in the treatment group at six time points: baseline (pre-stimulation) and five further time points, each performed following the cumulative stimulations (Figure 1). The sham group underwent a pre-test and was measured a second time after 420 pseudo-stimulations. All testing was conducted during a single experimental session lasting approximately 1½ hour. Identical foot positioning was carefully reconstructed for each measurement timepoints to ensure consistency in biomechanical conditions during reflex elicitation.

All procedures were conducted in accordance with international ethical standards for sports science research [14]. Participants provided informed consent and were advised to abstain from alcohol consumption for 24 h prior to testing to avoid confounding effects on reflex excitability.

### 2.3. Electromyography and Recording

Surface EMG signals were recorded from the soleus muscle using cup electrodes (HELLIGE Baby electrodes, Ag/AgCl, approximately 12 mm^2^ surface area). Electrodes were positioned laterally along the soleus muscle belly, oriented parallel to the predominant longitudinal fibre direction, with a standardized inter-electrode distance of 20 mm. A reference electrode was placed over electrically neutral tissue. Prior to electrode application, the skin was prepared through a standardized protocol: hair removal, cleaning with 70% alcohol, and light abrasion with fine-grit sandpaper to reduce skin impedance below 5 kΩ. Conductive electrode gel (HELLIGE) was applied to ensure optimal skin–electrode contact. Electrodes were secured with adhesive tape to prevent displacement during measurements. EMG signals were amplified using a custom-built differential amplifier (Biovision, Wehrheim, Germany) (gain ×1000, input impedance 16 GΩ, common-mode rejection ratio > 100 dB). Signals were digitized at 10,000 samples/s using a 12-bit analog-to-digital converter (DAQ-Card 6024, National Instruments, Austin, TX, USA). Data acquisition was controlled using DIAdem 8.1 software (National Instruments). Offline processing included baseline correction and removal of movement artifacts using a first-order Butterworth high-pass filter with a 1 Hz cut-off frequency, which effectively removed slow signal drift while preserving CMAP waveform characteristics [15].

### 2.4. Experimental Setup and Tendon Reflex Administration

Participants were seated upright on a custom measurement chair with standardized joint angles: 90° at the knee, hip, and ankle joints. This positioning was maintained throughout all measurements to ensure consistency. The left foot was secured to a foot plate using two Velcro straps. Both the thigh and lower leg were stabilized using frontal and distal fixation straps to minimize movement artifacts. Participants were instructed to maintain a relaxed, consistent posture with their arms and head in a fixed position throughout the experiment. Visual breathing instructions were displayed on a monitor to help participants maintain regular respiratory patterns.

Achilles tendon reflexes were elicited using an electrically driven reflex hammer (Copley Controls, Canton, MA, USA) mounted on a linear actuator. The system delivered highly reproducible impact forces with the following characteristics: maximum acceleration 407 m/s^2^, and maximum velocity 5.3 m/s. Prior to each measurement session, the hammer’s home position was calibrated by positioning it 40 mm from the Achilles tendon with a 7 N contact force threshold. This automated system ensured consistent impact parameters across all participants and measurement time points, critical for reliable CMAP amplitude comparisons. The actual impact force varied between 40 and 100 N depending on individual musculotendinous stiffness, but remained consistent within each participant across measurement sessions. The impact forces acting on the tendon triggers supra-maximal reflex responses [16]. Maximum impact force was achieved within 12 ms of contact. Minimal 12 reflexes were triggered per measurement timepoint with a minimal 10 s inter-stimulus intervals to avoid reflex habituation (post-activation depression).

### 2.5. Repetitive Peripheral Magnetic Stimulation Protocol

Magnetic stimulation was delivered using a MagPro 100+ stimulator (MagVenture, Skovlunde, Denmark). The stimulation coil (type MMC-140) featured a parabolic, convex–inward design optimized for deep nerve stimulation. This coil generates symmetric biphasic pulses with a 280 µs duration and a maximum magnetic flux density of 4.5 Tesla at 100% output. The optimal stimulation site was determined individually by delivering single test pulses. The position was found when the lowest stimulation intensity at this position could still cause a palpable contraction of the calf muscles compared to neighboring stimulation sites. The handle of the coil points vertically downwards, with the current flow from the distal to the proximal direction over the nerve [17]. Once the optimal position was identified (lowest intensity producing palpable contraction), the coil position was marked directly on the skin using a waterproof marker. The coil handle orientation and the marked reference point ensured reproducible positioning throughout all stimulation sequences. Before each new stimulation block, the coil was repositioned using anatomical landmarks and the skin marking. Stimulation consistency was verified by observing comparable muscle contraction patterns across sessions. The stimulation coil was held manually by trained experimenters and pressed firmly against the skin with consistent pressure to minimize coil-to-nerve distance variability. While manual positioning introduces the potential for minor variations, this approach allowed for anatomical adjustment to individual leg contours while the marked reference point and standardized handle orientation ensured positioning consistency.

To optimize activation of the tibial nerve, each participant stood in an upright position with their hands placed against a wall for support. The left knee was maintained in nearly full extension. The magnetic stimulation coil was firmly positioned in the upper region of the posterior crural area, located in the immediate vicinity of the tibial nerve. This positioning ensured that the magnetic field effectively penetrated the target nerve structure. When the stimulation was delivered, visible contractions of the gastrocnemius and soleus muscles were observed, producing plantar flexion and relaxation of the calf musculature, which confirmed successful nerve activation.

The experimenters provided detailed instructions and supervised the stimulation procedure throughout the entire session to ensure proper administration of the repetitive peripheral magnetic stimulation. The participants in both the treatment and control groups were blinded to the research objective and the stimulation parameters. It was also ensured that participants could not discuss the experiment or the stimulation itself.

### 2.6. Experimental Protocol

The stimulation protocol [18] was delivered as follows: The experimental group received magnetic stimulation at an intensity of 60% of maximum stimulator output, which corresponded to a current flow of 94 A/µs. This intensity aligns with protocols used in our previous studies and lies within the range commonly reported in the rPMS literature (typically 50–80% of maximum output), and no participants reported pain or discomfort at this intensity level. For clinical application, recording the motor threshold is necessary to individualize the stimulation intensity depending on the neuromotor disorders. The stimulation was delivered at a frequency of 5 Hz, equivalent to five pulses per second with a 200-millisecond interstimulus interval. Each stimulation sequence consisted of 15 stimuli per train, with 3 s rest intervals between consecutive trains. The participants underwent progressive stimulation with incrementally increasing volumes of 105, 210, 315, 420, and 840 total stimuli, which corresponded to 7, 14, 21, 28, and 56 trains, respectively. Importantly, the study employed a cumulative dose design. Each successive condition represented an additional increment in stimulation (e.g., the 210-stimulus condition included the initial 105 stimuli plus 105 additional stimuli). No washout intervals were implemented between stimulation blocks, as the design specifically investigated cumulative effects. This approach was necessary to address the research question regarding minimal effective dose, but introduces the limitation that later measurements reflect both higher total stimulus counts and longer elapsed time since baseline. Due to the progressive cumulative design, later measurement time points occurred at increasingly later times in the experimental session. Specifically, the Post-105 measurement occurred approximately after 5 min into the protocol, while the Post-840 measurement occurred approximately after 70 min. This sequential design was necessary for practical implementation but introduces a temporal confound. All measurements were performed immediately following each stimulation session. No follow-up measurements at later time points were conducted, precluding any assessment of effect persistence or temporal decay characteristics.

To partially address this limitation, we included a control group that underwent comparable measurements without active stimulation. However, the control group design (only two measurement time points) does not fully isolate temporal effects from dose effects. During the sham stimulation, the same procedure (subject position, coil positioning, sham stimulation duration) was conducted, which could not excite the nerves to depolarization but could trigger external acoustic output. The control group received sham stimulation at an intensity of only 1% of the maximum stimulator output, which was sub-threshold for producing any physiological effects, but could trigger external acoustic output. This group received a single treatment consisting of 420 stimuli delivered using the same temporal pattern as the experimental group. This design was chosen to verify that sub-threshold magnetic stimulation produces no physiological effects, the body positioning, and procedural aspects do not influence reflex excitability, and temporal factors over about 30 min do not cause systematic CMAP amplitude changes. A full 6-timepoint sham protocol was not implemented for practical reasons, as the null hypothesis predicts no changes at anytime point.

For the experimental group, each rPMS treatment was immediately followed by repositioning the participant in the measurement chair and recording of a minimum of 12 valid Achilles tendon reflexes (5 manual habituation taps followed by 20 measurement taps). The complete experimental protocol consisted of six measurement time points: baseline (pre-5 Hz) and five post-stimulation. The total experimental duration was approximately 75 min per participant.

## 3. Data Analysis and Statistics

Compound muscle action potential peak-to-peak amplitudes were extracted from filtered EMG recordings using a custom algorithm that identified global minimum and maximum values relative to the zero baseline. For each participant, CMAP values were obtained per measurement time point and averaged to yield a single representative value.

Statistical analyses were conducted using SPSS 28 (SPSS Inc., Chicago, IL, USA). Descriptive statistics (mean ± SD) were calculated for all measurement timepoints. One-way repeated-measures analysis of variance (rANOVA) tested for overall treatment effects in the experimental group [19]. The repeated-measures design inherently controls for inter-individual baseline differences by using each participant as their own control. While considerable inter-individual variability in absolute CMAP amplitude was observed, this variability reflects natural physiological differences and does not compromise statistical power for detecting within-subject changes, as the rANOVA partitions between-subject and within-subject variance explicitly. Given that rPMS protocols in the literature consistently report inhibitory (not facilitatory) effects on reflex excitability, and our directional hypothesis predicted amplitude reductions, we employed one-tailed paired *t*-tests for these comparisons between baseline and post-stimulation time points (directional hypothesis of amplitude reduction). Corrections for multiple post hoc comparisons employed the Holm–Bonferroni method. Partial eta-squared (η^2^_p_) values from the rANOVA were interpreted as η^2^_p_ > 0.01 small, η^2^_p_ > 0.06 medium, and η^2^_p_ > 0.14 large. Effect sizes were calculated using Cohen’s d for paired *t*-tests (d_rm_) [20,21], with 95% confidence intervals computed using the method described by Lakens [22]. According to the guidelines for paired *t*-tests, effect sizes were interpreted as d_rm_ > 0.20 as small, d_rm_ > 0.50 as medium, d_rm_ > 0.80 as large, and d_rm_ ≥ 1.20 as very large. For the sham group, a two-tailed paired *t*-test was applied to compare pre- and post-measurements. This analytical approach was appropriate given our primary research question, which concerned dose–response relationships within the experimental group, with the control group serving to verify the absence of sham effects rather than enable full factorial group comparisons. Statistical significance was set at α = 0.05. All data are presented as mean ± standard deviation.

## 4. Results

All participants completed the full experimental protocol without adverse events. No participants reported pain during magnetic stimulation, confirming the advantage of rPMS over transcutaneous electrical stimulation. The stimulation protocol (840 stimuli over approximately 5½ min) was well-tolerated by all participants, with no reports of discomfort. Pre-stimulation CMAP amplitudes in the experimental group showed considerable inter-individual variability, with absolute values ranging from 0.35 mV to 5.22 mV (mean 1.74 ± 1.09 mV).

### 4.1. Statistical Analysis of Treatment Effects—Experimental Group

Statistical analyses focused on within-group changes in the treatment group across measurement time points. Due to the incomplete longitudinal assessment of the sham group, inferential statistical comparisons between groups were limited and interpreted cautiously. Data from the sham group were analyzed descriptively and served to contextualize the findings observed in the treatment group.

**Figure 2:**
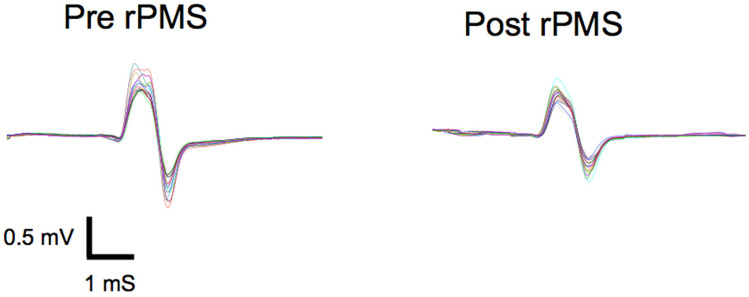
Representative raw CMAP traces from a single participant. Superimposed traces show baseline (**left**) and post-840 stimulation responses (**right**). Note the reduction in peak-to-peak amplitude with preserved latency.

Repetitive peripheral magnetic stimulation produced statistical significant and rapid reductions in soleus muscle reflex responses across all tested stimulation volumes. Figure 2 illustrates the CMAP amplitudes across the six measurement time points for the experimental group.

Mauchly’s test indicated that the assumption of sphericity was met (*p* > 0.209), and uncorrected degrees of freedom were reported. One-way repeated-measures ANOVA revealed a significant main effect of measurement time point (*F*(5, 80) = 4.98, *p* = 0.001, η^2^_p_ = 0.237, 90% CI [0.099, 0.349]), confirming that rPMS significantly altered soleus reflex excitability. This corresponds to Cohen’s d_rm_ = 1.13, 95% CI [0.54, 1.82], indicating a large-to-very large overall effect according to Lakens [22]. The omnibus ANOVA was evaluated at α = 0.05. Holm–Bonferroni correction was applied only to post hoc pairwise comparisons. (Table 1).

**Figure 3:**
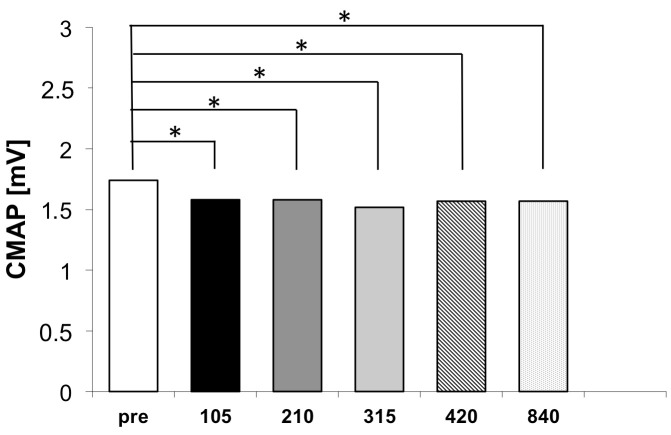
Changes in the amplitude of the compound muscle action potential (CMAP) of the soleus T-reflex following repetitive peripheral magnetic stimulation (rPMS) of the tibial nerve at different stimulation intensities. All post-intervention measurements differed statistically significantly from the pre-intervention baseline. Asterisks indicate significant differences from baseline (*p* < 0.05, Holm–Bonferroni-corrected). However, the differences between the various intensity conditions were not statistically significant.

Following the shortest stimulation protocol (105 stimuli), CMAP decreased from 1.74 ± 1.09 mV to 1.58 ± 1.15 mV, corresponding to a mean reduction of 0.16 mV. This change was associated with a large effect size (d_rm_ = 1.15, 95% CI [0.36, 1.94]), demonstrating that statistically robust reflex depression can be achieved with minimal stimulation volume (see Figure 3).

After 210 stimuli, CMAP remained unchanged at 1.58 ± 1.16 mV, yielding a mean reduction of 0.16 mV and a point estimate of the effect size in the moderate-to-large range (d_rm_ = 0.80), with a wide 95% confidence interval [0.05, 1.55] extending from very-small-to-large effects. Following 315 stimuli, CMAP further decreased to 1.52 ± 1.16 mV, resulting in the numerically largest mean reduction (0.22 mV) and a large effect size (d_rm_ = 1.02, 95% CI [0.23, 1.81]). However, the confidence intervals of the effect sizes substantially overlapped with those of the other stimulation volumes. After 420 and 840 stimuli, CMAP amplitudes were comparable (1.57 ± 1.06 mV and 1.57 ± 1.14 mV, respectively), with effect sizes (d_rm_ = 1.06, 95% CI [0.27, 1.85] and d_rm_ = 0.99, 95% CI [0.20, 1.78]) similar in magnitude to those observed after 105 stimuli. Importantly, no statistically or substantively larger effect sizes were observed with increasing stimulation volume, indicating that increasing the number of stimuli beyond 105 did not confer additional benefit.

When expressed in absolute terms, all post-stimulation CMAP values clustered tightly between 1.52 and 1.58 mV, compared to the baseline value of 1.74 mV. Critically, no statistically significant differences were detected among any of the five post-stimulation conditions (all pairwise comparisons *p* > 0.05). This finding indicates that the treatment effect plateaus rapidly and that stimulation volumes beyond 105 stimuli provide minimal incremental benefits within the immediate post-treatment assessment window.

Analysis of the dose–response relationship revealed several key findings. First, the onset of treatment effect was remarkably rapid, with a statistically significant CMAP reduction observed after just 105 stimuli (approximately 39 s of stimulation). Second, the numerically largest mean reduction was observed after 315 stimuli (approximately 117 s). However, the confidence intervals of the effect sizes overlapped substantially with those of the other stimulation volumes, indicating that this increase was not statistically or substantively greater. Third, increasing stimulation volume beyond 315 stimuli (to 420 or even 840 stimuli) did not yield additional benefit, with comparable effect sizes observed, confirming that maximal reflex inhibition was already achieved at lower stimulation doses.

### 4.2. Statistical Analysis of Sham Stimulation Group

The sham stimulation group, receiving placebo stimulation (420 stimuli), showed no statistical significant changes in reflex excitability. CMAP amplitudes remained stable, measuring 2.46 ± 1.49 mV before and 2.44 ± 1.44 mV after stimulation. A two-sided paired *t*-test revealed no significant difference between pre- and post-stimulation values (*p* ≤ 0.56), indicating that sham magnetic stimulation at 1% of maximum output had no effect on CMAP amplitude. In contrast (Table 2), the treatment group showed consistent CMAP reductions across all post-stimulation conditions (mean reduction: 0.16–0.22 mV, corresponding to 9–13% from baseline), whereas the control group demonstrated stability over a comparable timeframe. The magnitude of change in the experimental group was significantly greater than control group variability, confirming that the observed reflex suppression is attributable to active magnetic stimulation.

## 5. Discussion

This study demonstrates that low-frequency repetitive peripheral magnetic stimulation at 5 Hz produces rapid and statistical significant reductions in soleus muscle reflex excitability in healthy individuals. This exploratory study investigated the dose–response relationship of low-frequency (5 Hz) repetitive peripheral magnetic stimulation applied to the tibial nerve in healthy individuals. Our findings provide preliminary evidence that rPMS can produce measurable reductions in soleus muscle reflex excitability, with statistical and practical significance achieved in all tested stimulation volumes. The most striking finding is the small stimulation volume for a physiological effect, with statistical and physiological significant reflex depression observed after merely 105 stimuli delivered over 39 s.

Furthermore, increasing the stimulation volume to up to 840 stimuli did not result in any additional benefit from the treatment. These findings have important implications for optimizing clinical rPMS protocols, as they suggest that shorter treatment durations may be as equally effective as prolonged stimulation sessions, potentially improving patient compliance and clinical efficiency.

This pattern differs from the linear dose–response relationships often assumed in clinical practice [2,23], and has considerable implications for treatment efficiency. The plateau observed beyond 105 stimuli indicates that there is no advantage to prolonged stimulation sessions. This finding may contradict the common clinical assumption that longer treatment durations necessarily produce greater therapeutic effects [12]. From a practical standpoint, this suggests that effective rPMS treatments could be delivered in less than 120 s, making the modality highly feasible for clinical implementation where time efficiency is paramount.

The observed reductions in CMAP amplitude across all active stimulation conditions correspond to very-small-to-large effect sizes, indicating a meaningful physiological change in spinal reflex excitability. These findings are consistent with an earlier study [24], which also reported a reduction in reflex responses of comparable magnitude, further supporting the robustness of rPMS-induced inhibition. The wide confidence intervals reflect the small sample size of this exploratory within-subject study and the substantial inter-individual variability in CMAP responses. The observed CMAP amplitude reduction represents a physiologically meaningful change in spinal reflex excitability. This magnitude is comparable to H-reflex modulations produced by voluntary motor preparation and falls within the range of naturally occurring spinal inhibitory mechanisms. However, interpreting clinical relevance requires caution. In our healthy participants with normal baseline reflex excitability, the observed reduction represents the modulation of an already optimally regulated system. In clinical populations with pathologically elevated reflexes, proportionally similar reductions might translate to functionally significant improvements in motor control, pain reduction, or range of motion.

The stimulation effects observed in this study align with previous reports of rPMS-induced reductions in muscle tone and spasticity; ref. [3] reported long-lasting depression of soleus motor neuron excitability following repetitive magnetic stimulation of the spinal cord in multiple sclerosis patients using 25 Hz stimulation. Struppler and colleagues [4] demonstrated that 20 Hz peripheral magnetic stimulation could improve motor function in stroke patients, with effects attributed to the normalization of hyperactive stretch reflexes. More recently, theta-burst stimulation patterns delivered at 5 Hz were used to successfully reduce spasticity in children with cerebral palsy [7]. The current findings extend this literature by systematically characterizing the minimal effective dose and demonstrating that physiological effects can be achieved with remarkably brief stimulation periods. This has implications for pediatric applications, where patient tolerance and cooperation limit treatment duration. If similar dose–response characteristics apply in clinical populations with spasticity, effective treatments could potentially be delivered in approximately one minute, greatly enhancing feasibility for regular therapeutic use.

Compared with transcutaneous electrical stimulation, rPMS offers the significant advantage of painless application. While both modalities can activate peripheral nerves and modulate spinal excitability, the pain associated with electrical stimulation, particularly at intensities necessary for deep muscle activation, limits patient acceptance and may introduce confounding effects through pain-mediated reflex inhibition. The absence of pain reports in the current study, despite the delivery of up to 840 stimuli, confirms the tolerability advantage of magnetic stimulation and supports its potential for widespread clinical adoption.

Several methodological aspects of this study warrant discussion. The use of automated reflex hammer triggering with precise force control represents a substantial strength, eliminating examiner-dependent variability that plagues manual reflex testing. The quantification of reflex responses through CMAP amplitude measurement provides objective, continuous data superior to ordinal clinical scales. However, CMAP amplitude reflects the compound electrical activity of motor units and may not fully capture changes in higher motor circuits. The study population of healthy young adults limits generalizability to clinical populations with pathologically elevated muscle tone. Patients with spasticity due to upper motor neuron lesions may show different dose–response characteristics due to altered spinal circuitry, impaired descending inhibition, or changes in peripheral nerve and muscle properties. The magnitude of the effect needed for clinically meaningful improvements in function may differ substantially from the physiological changes observed in healthy individuals. Replication studies in patient populations are essential to validate clinical utility.

The immediate post-treatment assessment window employed in this study provided only a snapshot of rPMS effects. Clinical applications require sustained effects lasting from hours to days to be functionally meaningful [25]. Whether the 11–14% reflex reduction observed immediately post-treatment translates to sustained changes in muscle tone and whether higher stimulation volumes produce more durable effects remains unknown. Longitudinal studies with multiple assessment time points extending over hours and days post-treatment are needed to characterize the full temporal profile of rPMS effects.

The stimulation parameters employed in this study, particularly the 60% intensity setting, were chosen based on prior experience [3] to reliably activate the tibial nerve while remaining comfortable for participants. However, the optimal stimulation intensity likely varies based on individual factors, including subcutaneous adipose tissue thickness, nerve depth, and tissue electrical properties. The 5 Hz stimulation frequency was selected based on previous work [6,24], suggesting that low-frequency repetitive stimulation produces inhibitory effects on neural circuits. The theoretical basis for this frequency selection derives from studies of repetitive transcranial magnetic stimulation, where frequencies below 5 Hz generally produce suppressive effects on cortical excitability while higher frequencies produce facilitatory effects [11,26]. Whether this frequency-dependency extends to peripheral nerve stimulation and whether other low frequencies might produce superior effects remains to be systematically investigated. Identifying predictors of treatment response could enable stratified or personalized treatment approaches.

### Implications and Limitations for Clinical Translation

The translation to clinical populations with elevated muscle tone is paramount. Patients with spasticity due to stroke, spinal cord injury, multiple sclerosis, or cerebral palsy represent the target populations for therapeutic rPMS application. Dose–response studies in these populations will determine whether the plateau observed in healthy individuals also characterizes pathological states or whether higher doses provide a benefit when baseline excitability is markedly elevated. In clinical populations, particularly those with demyelinating diseases (e.g., multiple sclerosis) or peripheral neuropathies, comprehensive CMAP analysis, including latency and waveform morphology, may provide additional insights into disease-specific mechanisms. However, for investigating fundamental dose–response relationships of rPMS in healthy individuals, amplitude remains the most appropriate and informative primary outcome measure.

Finally, investigation of optimal treatment schedules for sustained therapeutic benefit is needed. Single-session effects, while physiologically interesting, have limited clinical utility unless they translate to lasting functional improvements. The findings of this study open several important avenues for future investigation. A systematic exploration of the temporal dynamics of rPMS effects is needed, including both the onset kinetics during stimulation and the decay kinetics post-treatment. Understanding whether effects accumulate during prolonged stimulation sessions or show rapid saturation would inform optimal treatment scheduling.

## 6. Conclusions

This study indicates that low-frequency repetitive peripheral magnetic stimulation produces rapid, significant reductions in spinal reflex excitability with remarkably low stimulation volumes. The treatment effect plateaus early at approximately 105 stimuli delivered over 39 s, with no additional benefit from higher doses in the immediate post-treatment period. These findings, reported in accordance with CONSORT guidelines, provide preliminary dose–response data regarding the immediate effects of rPMS treatment. These results suggest that effective treatments may be delivered much more efficiently than previously assumed, reducing patient burden and treatment time. The painless, non-invasive nature of rPMS combined with its demonstrated efficacy in modulating reflex excitability positions this modality as a promising therapeutic approach for managing elevated muscle tone in healthy humans. Further research in clinical populations with longitudinal follow-up is warranted to fully characterize the therapeutic potential and optimal application protocols for rPMS in neurorehabilitation.

## Figures and Tables

**Table 1:** Descriptive statistics and Holm–Bonferroni-adjusted pairwise comparisons for CMAP [mV] volume across stimulus conditions.

Stimulus	MQ [mV] ± *SD*	95% CI for *M*	Pre vs. Cond	Mean Diff	SE	*p*	Cohen’s d_rm_ (95% CI)
pre	1.74 ± 1.09	[1.18, 2.30]	–	–	–	–	–
S105	1.58 ± 1.15	[0.99, 2.17]	pre > S105	0.160	0.042	0.004	1.15 [0.36, 1.94]
S210	1.58 ± 1.16	[0.99, 2.17]	pre > S210	0.160	0.056	0.003	0.80 [0.05, 1.55]
S315	1.52 ± 1.16	[0.92, 2.12]	pre > S315	0.223	0.068	0.001	1.02 [0.23, 1.81]
S420	1.57 ± 1.06	[1.02, 2.12]	pre > S420	0.172	0.049	0.007	1.06 [0.27, 1.85]
S840	1.57 ± 1.14	[0.98, 2.15]	pre > S840	0.172	0.051	0.001	0.99 [0.20, 1.78]

**Table 2:** Comparison between experimental group and control group.

Parameter	Experimental Group	Control Group
Baseline CMAP (mV)	1.74 ± 1.09	2.46 ± 1.49
Post-treatment CMAP (mV)	1.52–1.58 *	2.44 ± 1.44
Absolute change (mV)	−0.16 to −0.22	−0.02
Relative change (%)	−9.2 to −12.8	−0.8
Effect size (Cohen’s d)	0.80–1.15	0.01
Statistical significance	*p* < 0.007	*p* = 0.56

* Range represents five post-stimulation time points (105–840 stimuli).

## Data Availability

The data that support the findings of this study are not publicly available because the author do not have access to secure, long-term repository infrastructure that would allow compliant online data management and sharing. However, the data are available from the corresponding author on reasonable request.
